# Supplementary figures and images for: An Alba-domain protein required for proteome remodelling during trypanosome differentiation and host transition
Source: PLoS Pathog. 2021 Jan 25;17(1):e1009239. doi: 10.1371/journal.ppat.1009239 (PMC7861527; doi:10.1371/journal.ppat.1009239)

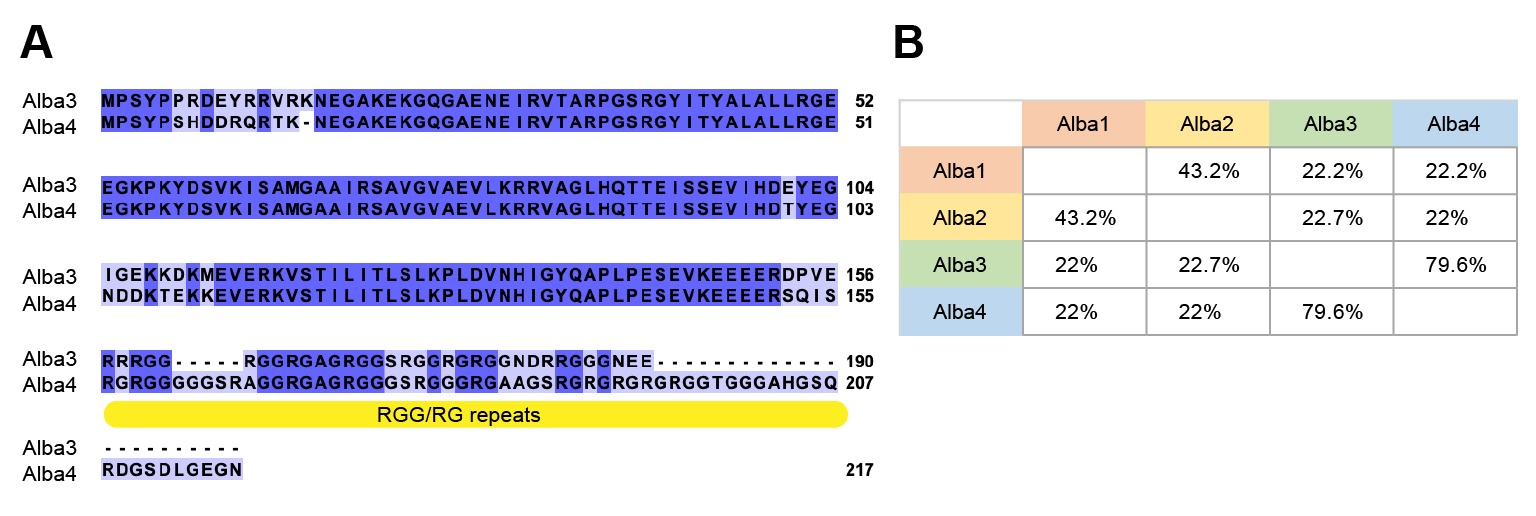

Supplement: S1 Fig — Relationship of Alba proteins (A) ClustalW protein sequence alignment of Alba3 and Alba4. Dark blue regions indicate sequence identity. The yellow bar indicates the RGG/RG repeats present in the C-terminal regions. (B) Percentage protein identity between pairs of T. brucei Albas determined by using Clustal Omega pairwise alignment matrix [88,89]. (TIF) [file ppat.1009239.s001.tif]

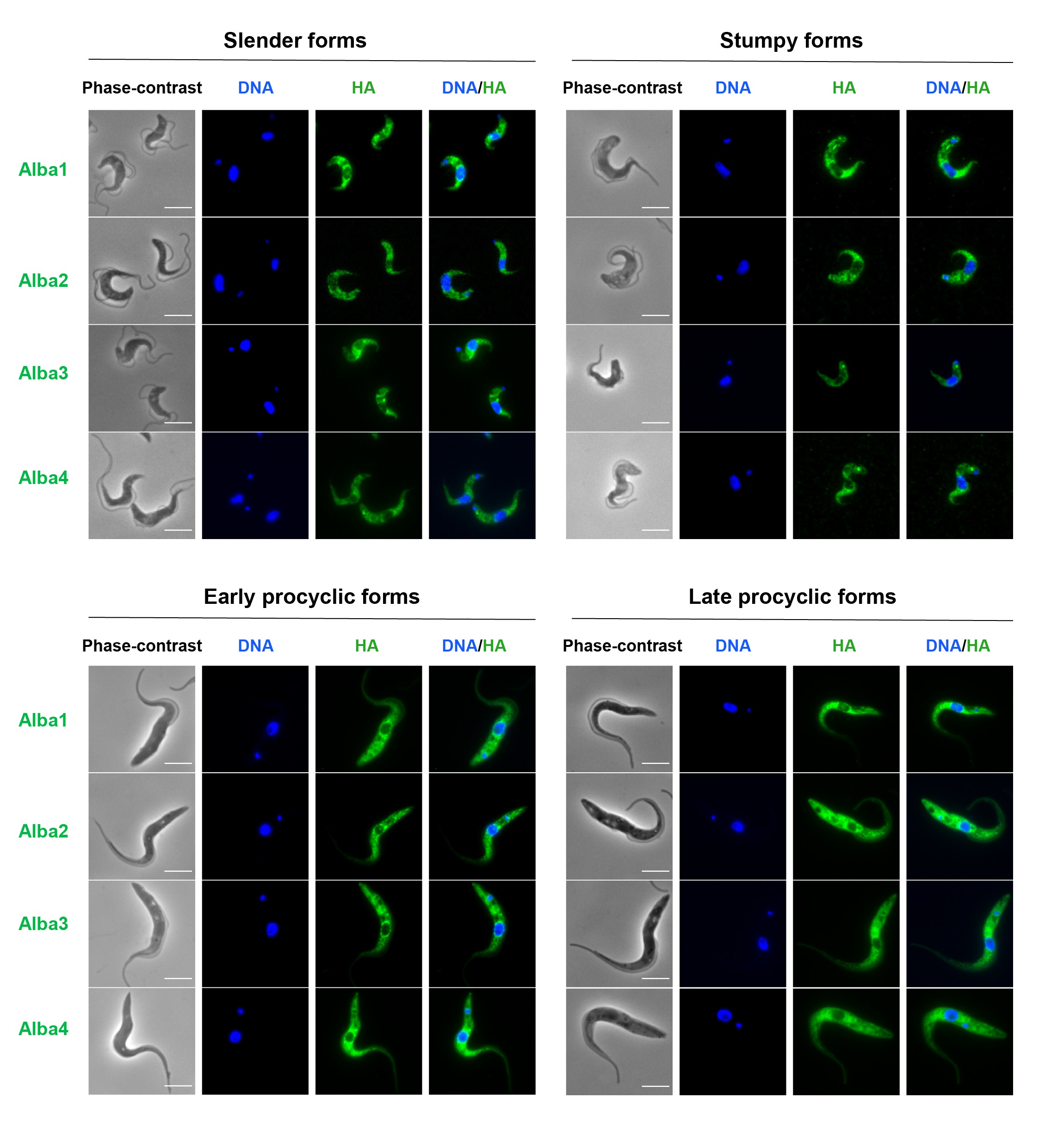

Supplement: S2 Fig — Cells were incubated with anti-HA. Scale bar, 5μm. (TIF) [file ppat.1009239.s002.tif]

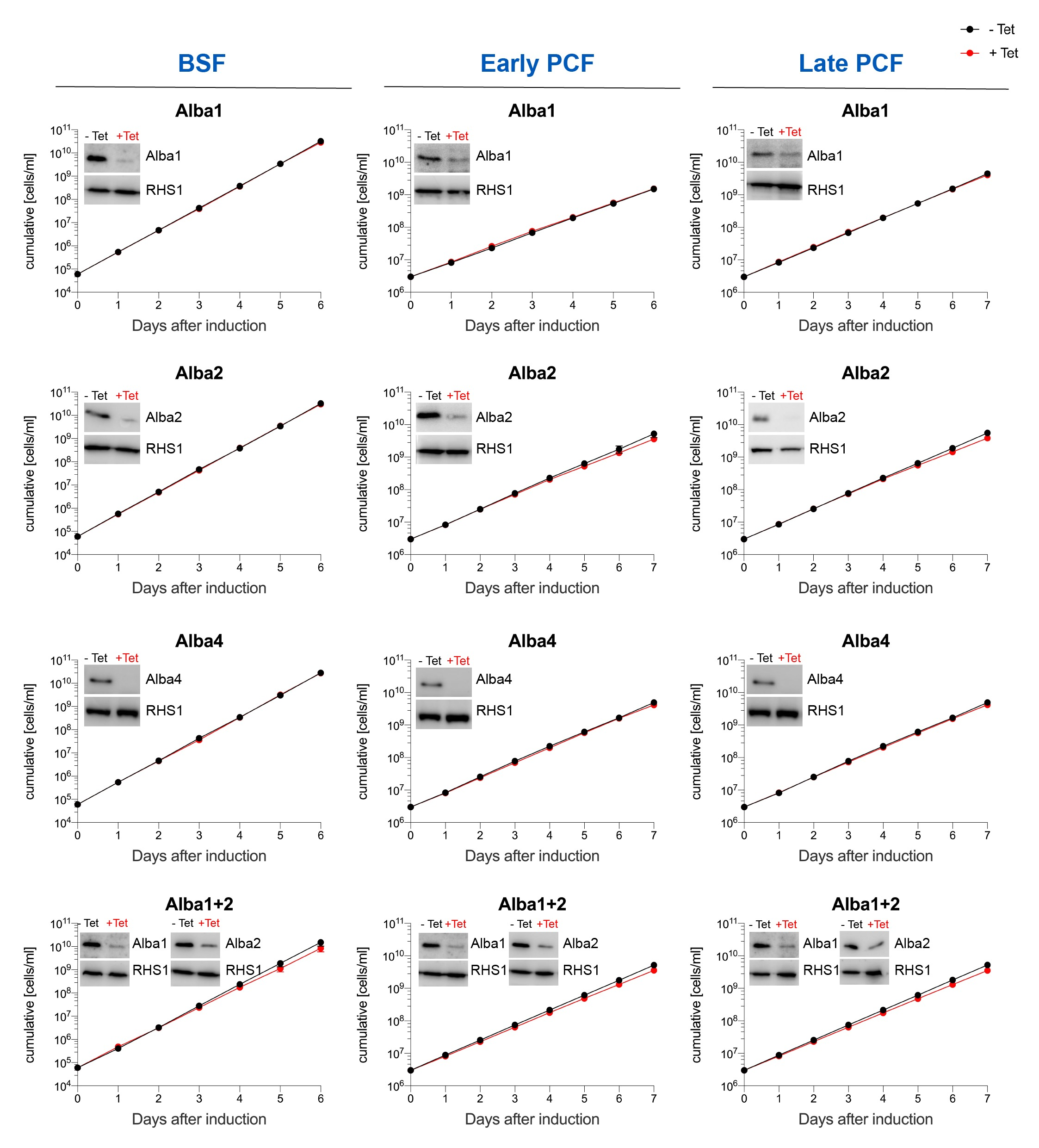

Supplement: S3 Fig — RNAi was performed in slender bloodstream forms (BSF), early procyclic forms (early PCF) and late procyclic forms (late PCF). Cumulative growth of cells was monitored for 6 days without RNAi induction (-Tet) or with RNAi induction (+Tet) by tetracycline. Error bars, mean ±SD (n = 3). Efficiency of knockdown by RNAi was assessed by Western blot analysis on day 4 after RNAi induction. RHS1 served as a loading control. (TIF) [file ppat.1009239.s003.tif]

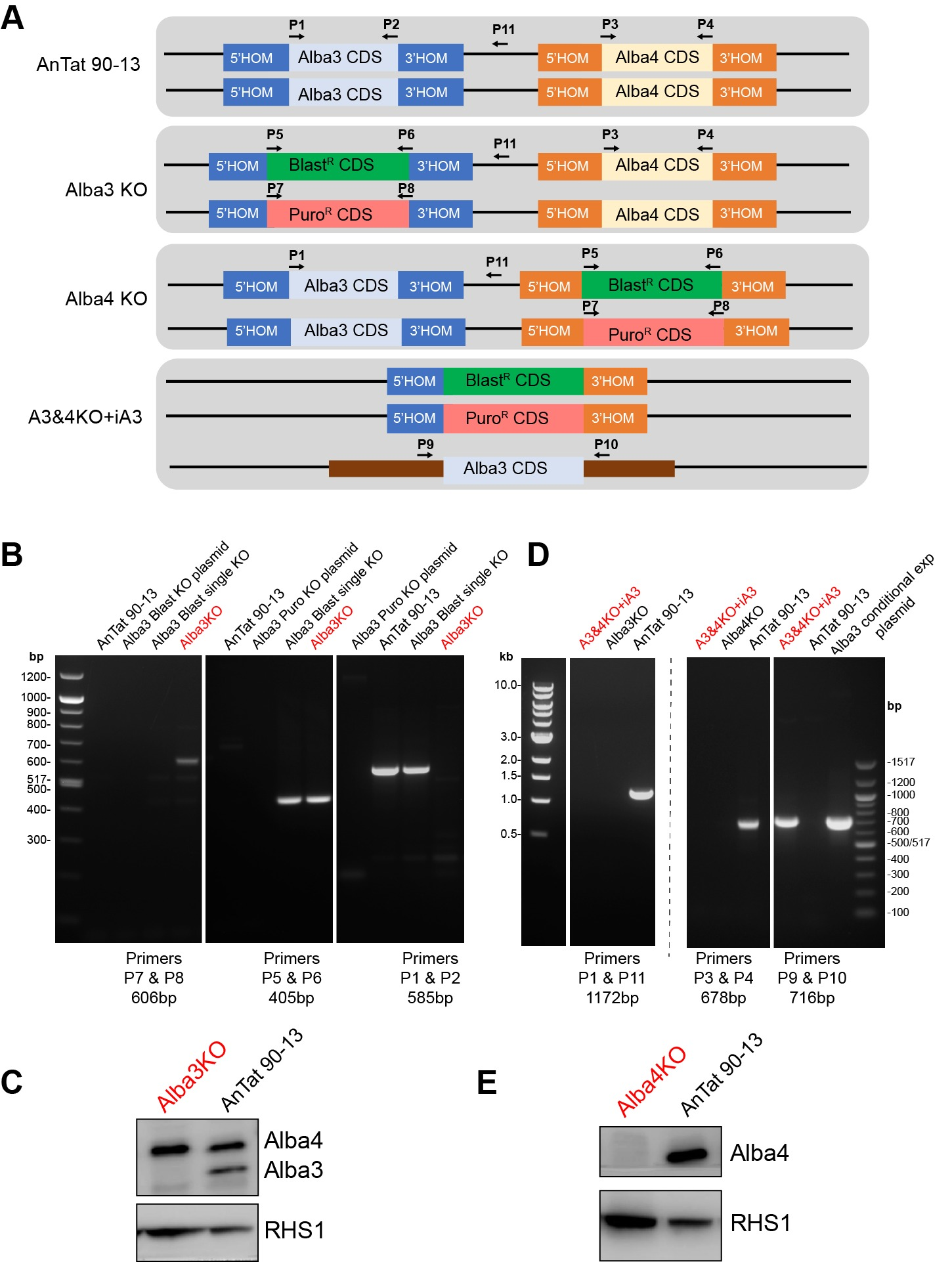

Supplement: S4 Fig — (A) Schematic representation of Alba 3+4 loci. (B) & (D) PCR confirmation of knockouts in Alba3KO, Alba4KO and Alba3&4KO+inducible Alba3 expression (A3&4KO+iA3) cell lines. Genomic DNA was isolated from individual cell lines and PCR was performed using primers indicated in the diagram in (A). (C) & (E) Western blot analyses of Alba3KO and Alba4KO. RHS1 served as a loading control. HOM, homology arms; CDS, coding sequence; BlastR, blasticidin resistance gene; PuroR, puromycin resistance gene. The sequences of the numbered primers (P1-11) are given in S4 Table. (TIF) [file ppat.1009239.s004.tif]

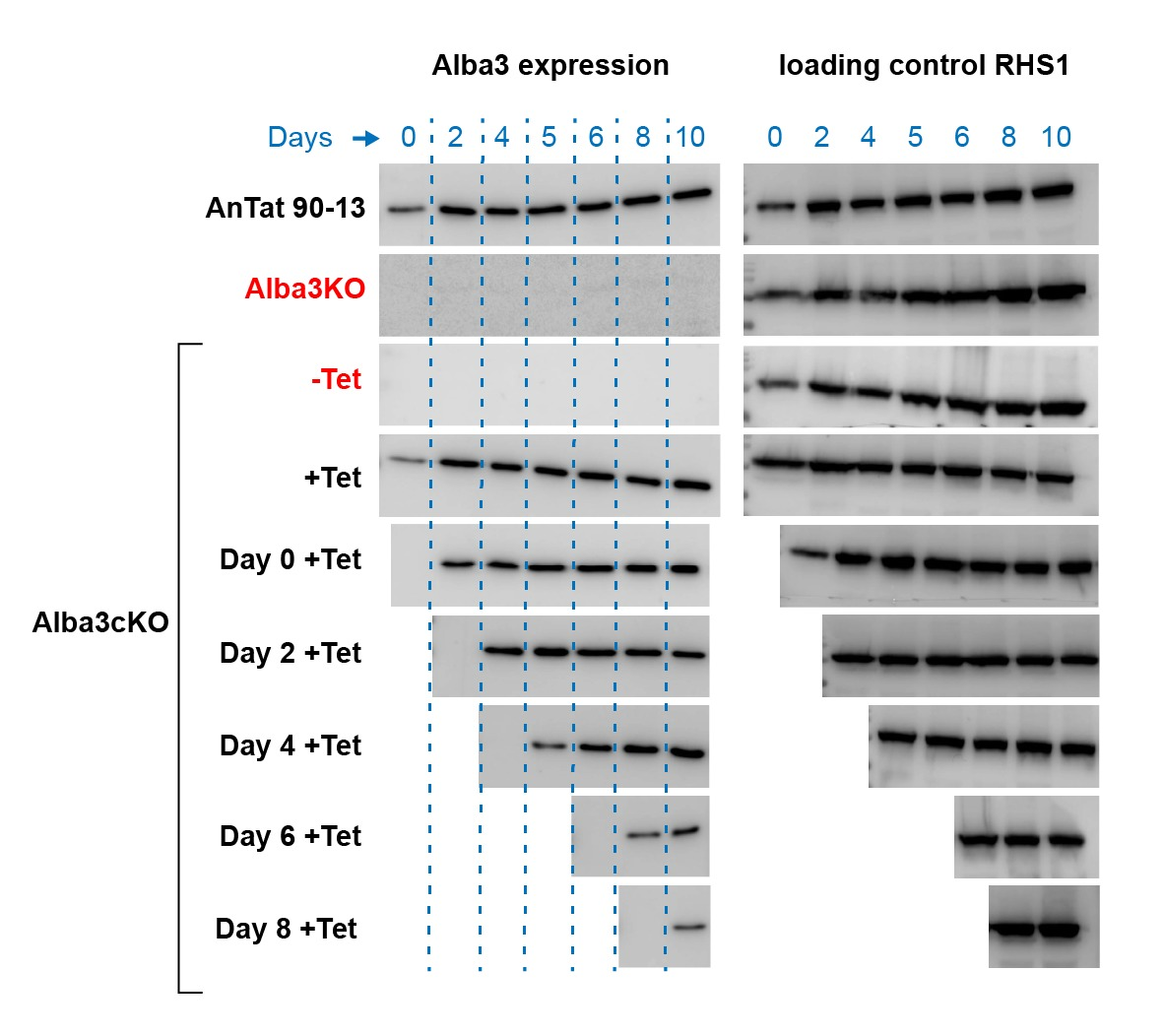

Supplement: S5 Fig — Western blot analysis of Alba3 expression in AnTat 90–13, Alba3KO and Alba3cKO (-/+Tet) during differentiation. Alba3 expression was induced in Alba3cKO at different time points from the start of differentiation. RHS1 served as a loading control. (TIF) [file ppat.1009239.s005.tif]

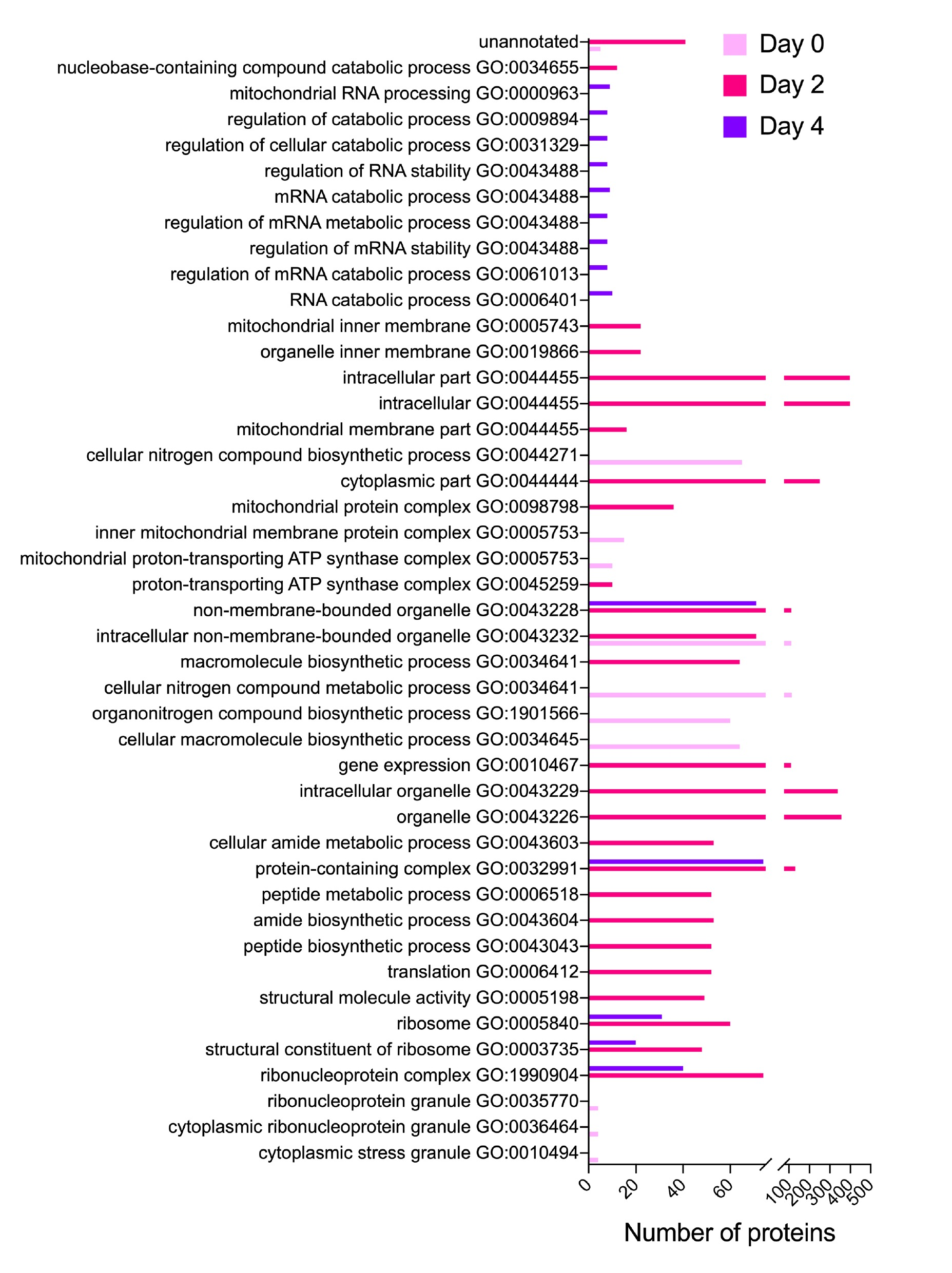

Supplement: S6 Fig — GO term analysis of significantly down-regulated proteins (p value <0.01, fold change ≥ 2) in Alba3KO compared to AnTat 90–13 on days 0, 2 and 4 of differentiation. The x-axis represents the number of proteins and the y-axis shows GO categories. (TIF) [file ppat.1009239.s006.tif]

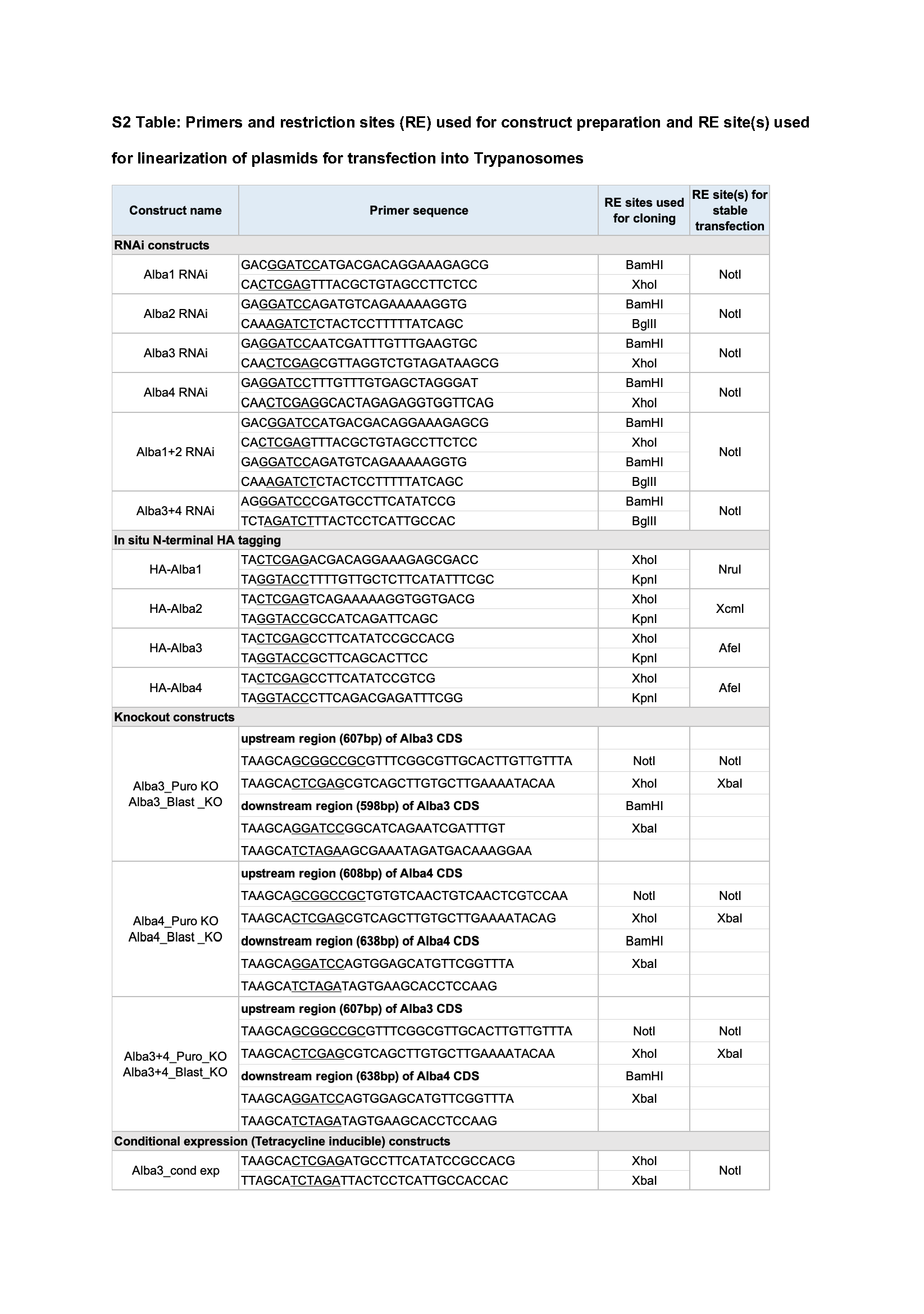

Supplement: S2 Table — (TIFF) [file ppat.1009239.s008.tiff]

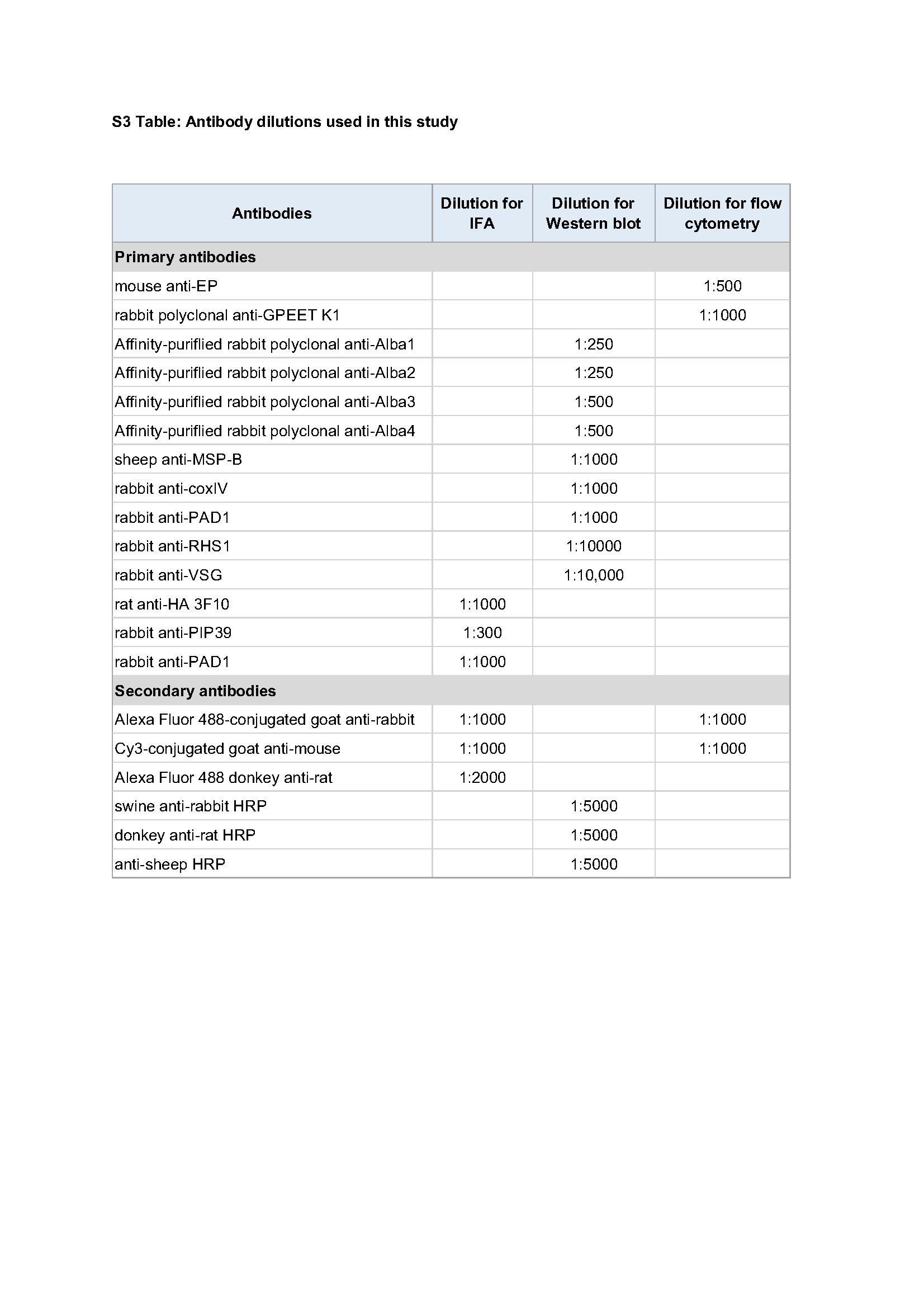

Supplement: S3 Table — (TIF) [file ppat.1009239.s009.tif]

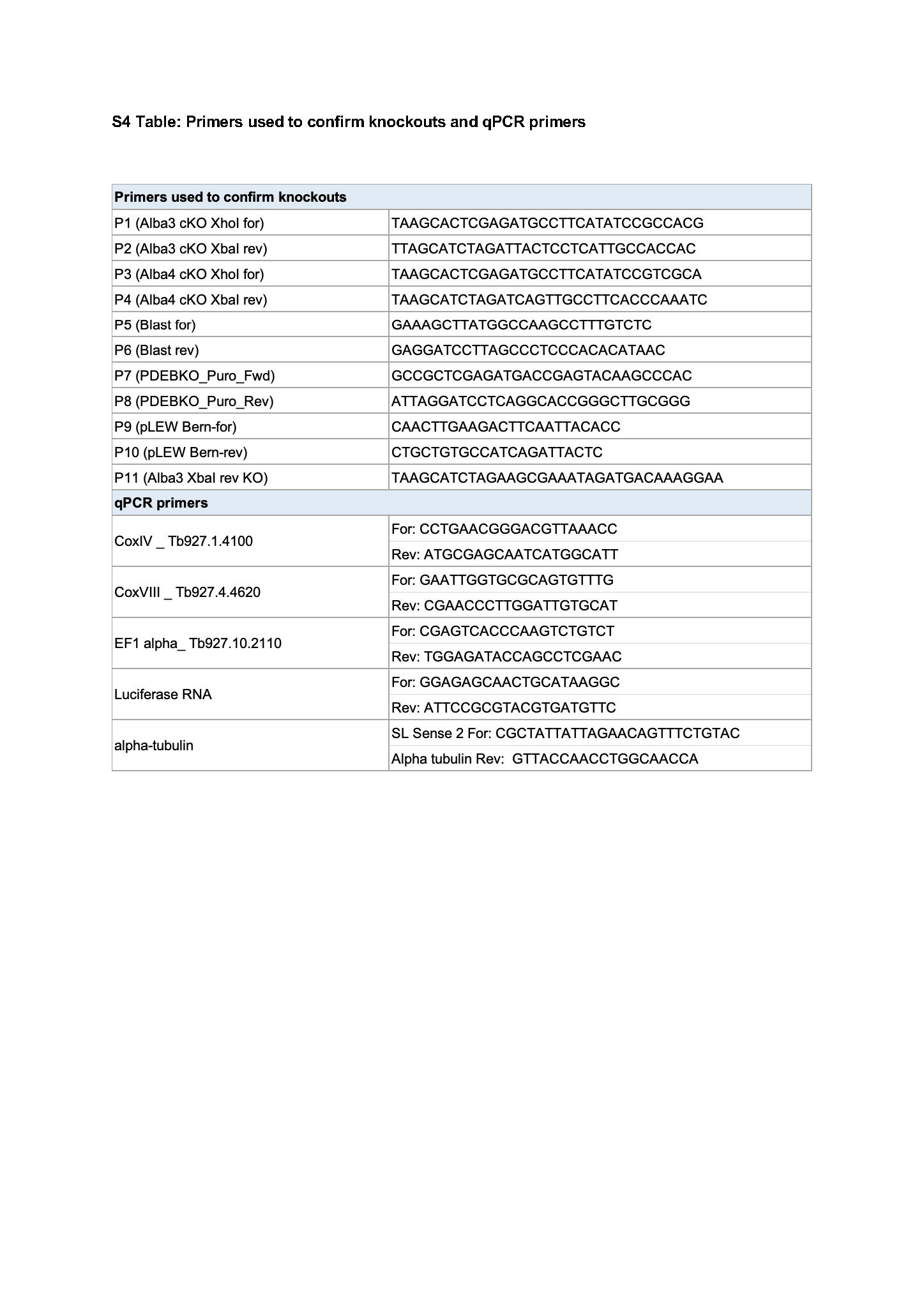

Supplement: S4 Table — (TIFF) [file ppat.1009239.s010.tiff]
