# Supplementary material for: An Alba-domain protein required for proteome remodelling during trypanosome differentiation and host transition
Source: PLoS Pathog. 2021 Jan 25;17(1):e1009239. doi: 10.1371/journal.ppat.1009239 (PMC7861527; doi:10.1371/journal.ppat.1009239)
Supplement: Attachment — Submitted filename: Alba 2020_PLOS rebuttal.docx [file ppat.1009239.s012.docx]

Dear Editors,

Re: PPATHOGENS-D-20-01665

Thank you for the reviewers’ constructive comments on our manuscript. We are convinced that these revisions have enhanced the quality of the work. For simplicity, we have interpolated our answers into the letter you sent us. Line numbers refer to the tracked version of the manuscript.

We hope that the modifications now make the manuscript acceptable for publication in PLOS Pathogens.

Yours sincerely,

Isabel Roditi

-------------

**Section 1**

**a.** the phenotype of Alba3 knockout cells during the time-course of differentiation to procyclic forms: where do they arrest- in the first cell cycle, or thereafter?

- Additional experiments (new Figure 2D) - show that Alba3KO has a slight delay in re-entering the cell cycle, but it does so, and divides exponentially for the first four days, albeit with a longer population doubling time than its parent. The text (lines 174-178) has been modified to incorporate these data.

Is VSG lost with normal kinetics? This will help position the point at which Alba 3 is important in differentiation.

- We were fortunate on two counts; first, RNA-seq established that both the parental line and the Alba3KO expressed VSG AnTat1.1 and second, we were able to obtain antibodies from Jay Bangs. Western blots showed that Alba3KO lost VSG with a delay of 24h compared to the parental line. Figure 4B (upper panel) shows one of three representative experiments. The text (lines 219-221) has been modified to incorporate these data.

**b.** How fly infectivity and parasite progression develops over time in the tsetse fly and how this relates to the kinetics of the differentiation failure in vitro?

- We conducted an additional fly infection experiment that ran for 28-30 days. The reason for choosing this time point was so that we could also assess salivary gland infections. This experiment shows that Alba3KO does not “catch up” over time. On the contrary the infection rate drops. This experiment has been included in the manuscript (Figure 3C and lines 204-210).

**c**. Confirmation that the protein synthesis assay used is consistent with more conventional assays using 35S methionine

- These amino acid analogues have been very widely used in organisms ranging from bacteria to mammals, including several parasites (e.g. *Leishmania*, *Toxoplasma*). There have also been comparisons between ^35^S Methionine and analogue incorporation with respect to mitochondrial proteins. These have shown to be equivalent. A few references and a link to the data sheet are included here:
- https://doi.org/10.1101/2020.07.22.215194
- https://doi.org/10.1371/journal.pntd.0007651
- https://doi.org/10.1101/2020.07.22.215194
- https://doi.org/10.1371/journal.pntd.0007651
- https://doi.org/10.1371/journal.ppat.1005927
- https://doi.org/10.3389/fmars.2014.00048
- https://doi.org/10.1186/s13036-019-0166-3
- https://doi.org/10.1038/srep32377
- https://doi.org/10.1038/s41467-019-10542-0
- Data sheet- https://www.thermofisher.com/order/catalog/product/C10102#/C10102

**d.** Consideration of the ribosomal profile and biosynthesis in different life cycle stages and in the Alba 3 KO

- We have made the following considerations and incorporated them into the discussion (lines 426-440):

First, we confirmed that stumpy forms are translationally quiescent, as has been shown previously. Second, Alba3 is unlikely to be directly involved in ribosome biosynthesis as it is never detected in the nucleolus. Third, the Alba3 KO is still able to assemble ribosome subunits and monosomes. These formed the dominant peaks for the KO on days 2 and 4 of differentiation to procyclic forms. Fourth, we show that Alba3 is associated with polysomes during differentiation, but we cannot say for certain whether it associates with all polysomes or only a subset. Finally, we cannot conclude whether it is directly involved in polysome formation or if the effect is indirect.

**e.** Statistical analysis of the results to support your findings and to allow replacement of phrases that do not have scientific meaning with those that do.

- We have performed statistical analyses of fly infections and growth curves and incorporated these in the text and figures. The text has been modified to reflect whether differences were statistically significant or not.

**Section 2**

**Part I - Summary**

Reviewer #1: The T. brucei genome encodes 4 different Alba domain containing proteins, Alba1 to 4. Two of these, Alba 3 and 4, also contain a nucleic acid binding motif. The four Albas have previously been shown to localise to the cytoplasm of procyclic forms. The experiments in this manuscript start with an investigation of the subcellular localisation in bloodstream forms and make the discovery that Alba3 and 4 are concentrated in the Stumpy Regulatory Nexus in stumpy bloodstream forms. This finding suggests that they may play a role in the differentiation of mammalian bloodstream forms to insect procyclic forms and this was tested using first RNAi and then gene deletion. Knockdown of Alba3 produced a growth defect in procyclic forms but not in bloodstream forms, the other Albas did not and the remaining experiments concentrate on Alba3.

As bloodstream Alba3 -/- cells were fine in culture and in a murine model infection but stopped proliferating in differentiation to procyclic forms was induced in culture. This growth arrest was rescued by an inducible add back. These experiments indicate that Alba3 is involved for the differentiation to mature procyclic forms. Experimental infections of tsetse flies indicated that Alba3 was not necessary for differentiation and tsetse infection but Alba3-/- cells infected fewer flies and produced weaker infections.

Detailed analysis of the kinetics of differentiation showed that Alba3-/- cells were able to differentiate but did so more slowly than the parental. After differentiation, the procyclics forms stopped proliferating unless Alba3 was provided within 4 days of initiating differentiation.

Subsequent experiments investigated the molecular differences between parental and Alba3-/- cells. Mass spec comparison of the proteomes of parental and Alba3-/- indicated a reduction/absence of a large group of mitochondrial proteins in Alba3-/- cells. These differences were not reflected in mRNA levels suggesting a defect in translation.

Overall, the experiments in the paper provide strong evidence that Alba3 is necessary for the correct programme of translation on differentiation of stumpy bloodstream forms to procyclic forms and to establish a stable mature procyclic phenotype.

It is one of the first examples of a regulator of translation of a subset of mRNAs in tryps and will be of interest to a wide audience.

Reviewer #2: This manuscript details how two predicted RNA binding proteins in Trypanosoma brucei contribute to life cycle development. The Alba proteins in T. brucei have been previously discovered and characterised to some extent, but this paper carries out a thorough analysis of the importance of, particularly, Alba 3. This involves carefully executed experiments whereby Alba 3 expression is inducibly knocked down by RNAi, or full gene deletion achieved - with conditional re-expression. The resulting phenotypes and mRNA and proteome changes are characterised in the resulting lines, as are the detailed consequences for different stages of development. The quality of the experimental work and clarity of writing is characteristic of this group and the paper nicely shows that Alba 3 influences the efficiency of transition from early differentiated procyclic forms (i.e. soon after their generation from stumpy forms) to proliferative established early procyclic forms. The evidence supports their contention that Alba 3 affects proteome remodelling early in differentiation and that Alba 3 and Alba 4 show some functional redundancy in the bloodstream but not procyclic forms. Although direct versus indirect effects of Alba 3 on translation in early differentiation have not been pinned down, this is very difficult to establish and does not diminish the overall precision of the analysis reported. I have only a few requests for clarification and suggestions for improvement.

Reviewer #3: In this manuscript the authors characterize the family of Alba-domain proteins (Alba1-4) from Trypanosoma brucei. RNAi of each individual Alba genes show that they are not essential for growth. However, Alba3 appears to be important during the differentiation of stumpy cells into procyclics. This in vitro observation was then validated in vivo by infecting tsetse flies, which show a significant reduction in both the midgut and the proventriculus infection rates. The authors then show evidence that Alba3 is important to scape from translational repression, which they suggest impacts the formation of procyclic-specific mitochondrial respiratory complex proteins and the repression of some bloodstream-specific proteins.

The paper is well written and the strength of it is on the discovery that Alba3 is important for procyclic differentiation/colonization of the fly. However, two things concern me:

1) Some of the findings and conclusions of this paper are similar to the ones reported by the same group in 2011 (Mani J., et al. 2011 PLoS One), so I wonder about how much progress has been made and,

- In the previous paper, analysis of Alba proteins was restricted to procyclic forms. Here we also look at slender and stumpy bloodstream forms as well as differentiation to procyclic forms. We also include mouse and fly experiments and comprehensive proteomic and transcriptomic analyses. In the previous paper, this was restricted to 2DIGE. The previous paper used only RNAi; here we also made knockouts and conditional knockouts. The finding that Alba3 is required for proteome remodelling during differentiation is also completely new.

2) the finding that Alba3 is important for the transition between stumpies and PCF is exciting, but I feel that the Alba3 KO mutants should have been better characterized both in vitro and in vivo.

- We analysed multiple differentiation markers and include new data in this revised version (VSG loss and cell-cycle re-entry), we performed polysome profiling and proteome analysis on different days. We also characterised the behaviour of the Alba3KO mutant in mice and in tsetse flies (now for up to 30 days).

For instance, why do the cells stop growing in vitro (defects in cell cycle?)?

- Since there are massive effects on the proteome, it is difficult to attribute growth arrest to one cause.

Do they have problems with motility, flagellum structure or organelle repositioning after differentiation?

- We examined kinetoplast re-positioning in the original version of the manuscript and showed that there was a slight delay. We did not look at motility because the KO cells were already highly compromised by the lack of mitochondrial proteins.

Likewise in the fly, the infections look lower in the Alba3 KO, but do they catch up with WT levels if scored later, for instance, at 21 dpi?

- This has been dealt in the reply to the editor section 1. The Alba3KO gives even fewer midgut and proventriculus infections at later time points and no salivary gland infections (days 28-30 post infection).

Furthermore, any evidence of a SoMo phenotype given that PV infections are significantly lower?

- It is not possible to test this. SoMo is exhibited by actively dividing/ growing early procyclic forms. Alba3KO parasites stop dividing by day 5 after differentiation to procyclic forms.
- Not all defects in fly infection manifest themselves as SoMo defects. For example, a knockout of MAP kinase kinase 1 (MKK1) completely abolished its ability to infect the salivary glands, while having no effect on midgut infections or SoMo [ Imhof, S., et al. 2014, Morand, S., et al. 2012 ]. Similarly, a knockout of the transmembrane protein PSSA-2 showed impaired colonisation of the glands, but normal SoMo and midgut infections [Imhof, S., et al. 2014, Fragoso. C.M., et al. 2009].

Last, but not least, why does the infectivity of the Alba3 AB cells are not included in this experiment?

- We did an experiment to ensure that Alba3 addback rescued the phenotype in flies. However, this was at a point when we had low infection rates generally and we prefer not to include this. It shows clearly, however, that (a) tetracycline does not affect AnTat 90-13 and (b) there is partial rescue of the midgut and proventriculus infections. In our experience it is normal that addbacks rescue partially and not completely.

­­

**Part II – Major Issues: Key Experiments Required for Acceptance**

Reviewer #1:

1. Is there an inconsistency between the tsetse infection which in some flies goes on for >4 days and the in vitro differentiation which stops proliferation?

- There is no inconsistency. While cells stop dividing in vitro, they do survive. Our experience, over the course of many years, is that there is variability between flies and some are more permissive than others.

2. Figure 6A
Does Figure 6A infer that stumpy forms contain very few ribosomes as well as very few polysomes and the subunits and monosomes peaks are very small? In the methods equal numbers are cell equivalents are used yet the y-axis for stumps has a different scale. These observations should be compared with previous findings (for example doi: 10.1371/journal.pone.0067069) The y-axes should be on the same scale

- We realise that we were not sufficiently clear in our description of these experiments. For technical reasons (presence of methyl cellulose in the culture medium of bloodstream forms, which meant resuspending the cells in a larger volume of polysome buffer), we were only able to load a smaller number of cell equivalents for stumpy forms on the gradients (3-fold fewer). We did, however, load the same number of cell equivalents for both the parental line and the KO. This is why all the peaks are smaller in these samples. Since differentiating cells were in DTM without methyl cellulose, more cell equivalents could be loaded on day 2 and day 4. In each case, the same cell equivalents were used for the parent and the KO. This is now clarified in the Material and Methods: lines 613-616 and in the legend of Figure 8A (lines 1078-1080)
- Our results are consistent with previous publications. Stumpy forms are translationally silent. Polysome profiles of slender and stumpy forms of AnTat1.1 were analysed previously by Brecht and Parsons, 1998 and also by Capewell et al., 2013. These studies revealed reduced or no polysome peaks in the stumpy forms.

 Brecht and Parsons, 1998

 Capewell et. al., 2013

It is a major finding if the decrease in protein synthesis is procyclics is due to a loss of ribosomes. Probably unlikely but if correct (as opposed to a technical cause for the observation), could Alba3 be delaying ribosome biosynthesis?

- We did consider that depletion of Alba3 might lead to a defect in ribosome biogenesis. It seems unlikely that Alba3 is directly involved as it is never localised to the nucleolus, which is the site of ribosome biogenesis. Moreover, the KO was still able to assemble ribosomal subunits and monosomes, which formed the dominant peaks.
- Although we referred to this in the original version of the discussion, it was probably too brief. We have now elaborated it to make this point clear (lines 426-440).

Could the number of repeats be added to the figure legends, for example the polysome analysis

- Figure 8A shows one of three biological replicates. This has been added to the figure legend.

3. Figure 6C
Has a comparison of the patterns of protein synthesis of L-HPG and 35S-methionine been reported? It is a minor point but I would like to be convinced they are the same.

- This has been dealt with in the reply to the editor in Section 1c.

Reviewer #2: None

Reviewer #3:

The co-localization of Alba3/4 with PIP39 in the STuRN is interesting, but this story kind of disappears from the paper and its significance is never discussed.

- Although we observed the additional localisation of Alba3 in stumpy forms early on in the project, its significance only became clear very recently (Szöőr et al, 2019). While it is interesting that regulators of stumpy to procyclic differentiation converge on the STuRN, it is not the main point of this study.

Does the delay in MSP expression in Alba3 KO cells suggests that removal of the VSG coat is also delayed in differentiating cells? Any evidence of that?

- We have now looked at VSG loss and confirmed that there is a delay in Alba3KO. This is dealt with in the response to the editor in section 1a.While this may be (partly) due to a delay in MSP B expression, GPI-PLC, which also participates in VSG shedding, is expected to act normally.

The fly infectivity of just WT cells is rather low (~40%) and so the comparison with Alba3 KO cells is difficult because of the overall low infection rate.

- For the flies that we get from Bratislava, we have had similar infection rates for the last decade at least (e.g., Vassella et al., 2009; Fragoso et al., 2009). This applies to both in vitro and in vivo generated stumpy forms. The Engstler lab gets similar infection rates with AnTat 1.1. Some other labs (e.g., Acosta Serrano lab) may have more permissive flies and/or more virulent strains of trypanosomes.

In addition, if flies were scored between 12-15 dpi it means that some variation in the infection phenotype can be expected considering the negligible growth in vitro of the KO cells. This also relates with my previous comment on the infection phenotype at 21 dpi and whether the infection level may eventually catch up over time.

- The KO does not “catch up” over time. This has been dealt with in the reply to the editor in section 1b.

**Part III – Minor Issues: Editorial and Data Presentation Modifications**

Reviewer #1: The starting cell line carries a couple of transgenes and should be referred to as ‘parental’ not ‘wild type’

- We have made the requested changes throughout the manuscript.

The authors use descriptors such as ‘critical’ and ‘pivotal’. These does not mean anything scientifically. They may consider using ‘necessary’ and ‘sufficient’ etc.

- The only time we have used the word ‘critical’ is in this sentence in the introduction: “The generation of preadapted transmission forms is a critical step in ensuring efficient parasite dissemination to the other host.” (The guest editor will probably agree with us).
- The word ‘pivotal’ was used once and it has now been replaced by ‘important’ in the revised manuscript.

Reviewer #2:

Line 51. Abstract (and elsewhere in the text): “We show that Alba 3 is key to escape from translational repression…”. This feels a little too strong given that translation resumes in Alba KO lines albeit with delayed kinetics. Perhaps tone down to something like “We show that Alba 3 is key to rapid/efficient escape from translational repression…”.

- We have made the modification suggested by the reviewer.

Line 100. The review reference cited is quite old- there are more recent examples (e.g. PMID: 31164043 from the same author).

- This has been replaced by a reference by the same author from 2019 and an additional reference.

Line 157 (and other places where quite subtle phenotypes are observed). Alba 3 KO creates slower growth of the cells- is this at the level of statistical significance? The effect seems clear but is subtle and so would benefit from statistical analysis. This is also true of several other assays in the manuscript where ‘representative’ results are shown from several biological replicates. There is inherent variability in developmental studies such as those reported and so it is OK to provide representative datasets, but there should be ways to normalise variability between datasets to provide a statistical/numerical assessment for treatment versus control groups.

- Statistical analyses have been performed and the p values have been incorporated in the revised manuscript.
- The differences in bloodstream form growth rates between the parental line and the Alba3 KO and Alba4 KO are not significant.

Line 160. It is intriguing that Alba 4 escapes RNAi in bloodstream forms when co-depleted with Alba 3. Could the authors speculate on how this might occur? The RNA machinery still clearly functions because Alba 3 is depleted and the failure to knockdown is always Alba 4, rather than either Alba 3 or 4 in different cells in the population.

- We have been racking our brains about this. The only thing that occurs to us is that Alba4 may be complexed with additional RNA-binding proteins and/or RNAs that might protect it in bloodstream forms. As we mentioned in the text, the same RNAi cell line knocked down Alba3 and Alba4 in procyclic forms.

Line 171 (Figure 2A-D) In the in vitro developmental studies it is reported and clear that stumpy are still generated in the Alba3 KO line – so, indeed, Alba 3 is not needed for stumpy formation. But it would be helpful to quantitate that since delayed or inefficient onward differentiation to procyclic forms could be generated if the level of stumpy forms was quantitatively less than in wild type cells. The data in Figure 3 suggest that stumpy formation is quite efficient, so it is not a major concern. Nonetheless, since other subtle phenotypes are reported in the paper, this easily quantifiable developmental step could be detailed.

- As suggested by the reviewer, we monitored the percentage of PAD1-positive cells (new Figure 2C). Both parental and KO stumpy forms were PAD-positive and the differences were not significant.

Line 182. Was the first cell cycle of the differentiating cells assessed? In other words, although kinetoplast repositioning clearly happens (albeit a bit delayed; Figure 3B), did the cells then progress to divide their kinetoplast and undergo mitosis? This would help to determine if the cells block after the first differentiation cell cycle is completed, or during the first cell cycle.

- This has been dealt with the answer to the editor (Section 1a) and a new Figure (2D) has been added to the manuscript.

Line 192-199. The infectivity of the Alba 3 KOs in tsetse flies is clearly less than wild type cells on average (Figure 3F). But nonetheless some flies establish medium and heavy midgut infections with the KO parasites. How do the authors explain this variability in the relative infectivity of the KO cells?

- A minority of flies became infected with the KO and sustained infections for up to 30 days. This most probably reflects heterogeneity in the fly population, with some being more permissive to infection than others. It does not imply that there is selection for a compensatory mutation in the KO. We observed previously that trypanosomes lacking procyclin genes infected the salivary glands at a lower rate than wild-type parasites. When the mutant parasites were passaged through mice, however, and used to re-infect flies, there was no increase in their salivary gland infectivity.
- This has now been incorporated into the discussion (lines 389-395).

Also please specify how many biological replicates were carried out- otherwise low infectivity to flies by the Alba3 KO could be a result of the particular bloodstock used (e.g. it contained less stumpies or was otherwise less infective for reasons unrelated to Alba 3).

- For the original manuscript, three fly experiments were performed. These were internally consistent. We have now added a fourth experiment which confirms these results.

Line 255. The difference between WT and Alba3 KO cells on day 2 and day 4 are reported not to overlap with the slender proteome described in Reference 24. It would have been helpful to have a parallel slender proteome dataset to the WT vs KO analyses derived from the same laboratory because interlab variation in proteomic studies will inevitably show differences. Are there any such slender datasets available as part of the same WT/KO experiment to strengthen the overall statement/argument?

- The Jansen proteome was also performed with AnTat1.1 (cultured in the same medium - HMI9 + methyl cellulose). We do not have our own data set for the proteome of slender forms.

Line 304-306 (Figure 6C) Why is the protein synthesis in the induced Alba3 re-expression so much stronger than in wild type cells?

- The short answer is that we do not know, but we observed this on day 2 in 3 out of 4 experiments. It was not due to transient overexpression of ectopic Alba3 (Figure S5).

Also, on Figure 6C it is reported tubulin levels are equivalent on day 2 and day 4 between the samples, but it seems a weaker in the day 2 samples with Alba3 KO without conditional rescue.

- The reviewer is correct that there is less tubulin on day 2 in the absence of ectopic Alba3. We have modified the text accordingly (lines 319-321). This now applies to Figure 8C.

Reviewer #3: None
